# *Ralstonia solanacearum* Infection Disturbed the Microbiome Structure Throughout the Whole Tobacco Crop Niche as Well as the Nitrogen Metabolism in Soil

**DOI:** 10.3389/fbioe.2022.903555

**Published:** 2022-06-21

**Authors:** Zhaobao Wang, Yuzhen Zhang, Guodong Bo, Yanping Zhang, Yu Chen, Minchong Shen, Peng Zhang, Guitong Li, Jie Zhou, Zhengfeng Li, Jianming Yang

**Affiliations:** ^1^ Energy-Rich Compounds Production by Photosynthetic Carbon Fixation Research Center, Shandong Key Lab of Applied Mycology, College of Life Sciences, Qingdao Agricultural University, Qingdao, China; ^2^ Tobacco Research Institute of Chinese Academy of Agricultural Sciences, Qingdao, China; ^3^ Grassland Agri-Husbandry Research Center, College of Grassland Science, Qingdao Agricultural University, Qingdao, China; ^4^ China Tobacco Yunnan Industrial Co., Ltd., Kunming, China

**Keywords:** nitrogen metabolism, microbial community, *Ralstonia solanacearum*, tobacco, rhizosphere soil, interaction

## Abstract

Infections of *Ralstonia solanacearum* result in huge agricultural and economic losses. As known, the proposal of effective biological measures for the control of soil disease depends on the complex interactions between pathogens, soil microbiota and soil properties, which remains to be studied. Previous studies have shown that the phosphorus availability increased pathobiome abundance and infection of rhizosphere microbial networks by *Ralstonia*. Similarly, as a nutrient necessary for plant growth, nitrogen has also been suggested to be strongly associated with *Ralstonia* infection. To further reveal the relationship between soil nitrogen content, soil nitrogen metabolism and *Ralstonia* pathogens, we investigated the effects of *R. solanacearum* infection on the whole tobacco niche and its soil nitrogen metabolism. The results demonstrated that *Ralstonia* infection resulted in a reduction of the ammonium nitrogen in soil and the total nitrogen in plant. The microbes in rhizosphere and the plant’s endophytes were also significantly disturbed by the infection. *Rhodanobacter* which is involved in nitrogen metabolism significantly decreased. Moreover, the load of microbial nitrogen metabolism genes in the rhizosphere soil significantly varied after the infection, resulting in a stronger denitrification process in the diseased soil. These results suggest that the application management strategies of nitrogen fertilizing and a balanced regulation of the rhizosphere and the endophytic microbes could be promising strategies in the biological control of soil-borne secondary disasters.

## Introduction

In agricultural production, plant pathogens can damage plant health and cause huge crop losses (McDonald and Stukenbrock, 2016). As some of the most significant plant diseases, plant-specific soil-borne diseases can seriously affect soil health and subsequent crop production. This is an ever-increasing threat and ungovernable problem on a global scale (Pérez-Brandán et al., 2014; Wei et al., 2019; Liu S et al., 2020). Bacterial wilt caused by *R. solanacearum* is a global disease that can infect most Solanaceae plants, such as eggplant, tomato, and tobacco (Peeters et al., 2013; Santiago et al., 2019). Researchers have been studying and analyzing the mechanism and searching for biological controls for *R. solanacearum* infection for many years. As reported, the extracellular polysaccharide (EPS) and the cell wall-degrading enzyme (CWDE) have been identified as major virulence factors (Genin and Denny, 2012), and *R. solanacearum* can promote the biosynthesis of these virulence factors by adopting the host plant’s metabolite L-glutamic acid during the course of the infection, resulting in the typical wilting symptoms of the host plant (Shen et al., 2020).

As the plant pathogen of a soil-borne disease, *R. solanacearum* infection is related to many factors, including the genetic and physiological properties of the pathogen, the physicochemical conditions of the soil, the structure of the microbial community of the rhizosphere, and the availability of some essential nutrients, such as phosphorus (P) and nitrogen (N) (Dalsing and Allen, 2014; Li P et al., 2021). In some work, the dominant strains in the rhizosphere and some microbial metabolites have been adopted to antagonize or inhibit the growth of *R. solanacearum*, resulting in the inhibition of *R. solanacearum* infection to some degree (Xue et al., 2013; Upreti and Thomas, 2015; Ge et al., 2017). Previous research has verified that phosphorus availability and microbial co-occurrence patterns synergistically affected pathobiome abundance, meanwhile, phosphorus availability increased the infection of rhizosphere microbial networks by *Ralstonia* in peanut crops (Li P et al., 2021). It is well known that soil microbes play a vital role in enhancing stress resistance, resisting soil pathogens, and regulating host immunity, and changes in soil physiochemical properties can inversely influence the microbial community of the rhizosphere (Jones et al., 2009; Mendes et al., 2013). The increase in endophytic beneficial bacteria of the rhizosphere and plant roots makes pathogen infection more difficult, thus making plants resistant to diseases (Achari and Raman, 2014; Wang et al., 2019; Mao et al., 2021). This highlights the joint function of the rhizosphere, the pathogens and the plants all play important roles in the plant’s resistance to pathogen infection. Moreover, soil microbes are closely related to the nutrient cycling in plant cultivation, including soil carbon (C) sequestration, greenhouse gas emissions, and soil nitrogen (N) conversion, among which nitrogen availability in soil is one of the limiting factors to the productivity of terrestrial ecosystems, and the process of soil nitrogen cycling is mostly driven by microbes (Howarth, 1991; Wallenstein and Vilgalys, 2005; Falkowski et al., 2008; Lebauer and Treseder, 2008; He et al., 2010; Pan et al., 2020). Because nitrogen plays an essential role in plant growth it is a common agricultural technique to fertilize crops with active and available nitrogen to remove nitrogen limitations on the crops, however this increase of active N within the soil has important impacts on the microbial communities of the soil (Fitter et al., 2005; Kermah et al., 2018; Fiorentini et al., 2019; Liu C et al., 2020). Moreover, nitrogen compounds and metabolism can take part in the plant pathogen infection process. Dalsing et al. verified that nitrate assimilation contributed to the process of root attachment, stem colonization, and virulence of *R. solanacearum* by deleting the gene encoding the catalytic subunit of *R. solanacearum*’s sole assimilatory nitrate reductase, *nasA* (Dalsing and Allen, 2014). Additionally, Ding et al. explored the interactions between tomato plants and two bacterial pathogens, *Pseudomonas syringae* and *R. solanacearum*, under four nitrogen regimes, revealing that nitrogen forms and metabolism affected the plants’ defense against pathogens in tomatoes (Ding et al., 2021). In summary, great efforts have been made to explore the mechanisms involved in *R. solanacearum* infection but the exact mechanism has yet to be discovered.

Furthermore, it is also not clearly understood how the *R. solanacearum* pathogen affects soil conditions, including the physicochemical properties, the microbial’s community structure, and the interaction between them. Therefore, this work analyzed *R. solanacearum*-infected soil and its corresponding tobacco samples (ST group) and compared them to healthy samples (CK group), which presented significant differences in many aspects, including the soil physicochemical properties, the plant’s nutrient contents, the microbial community of the rhizosphere, the endophytic microbial community within the tobacco plants, and the correlation between these factors. As a result, the abundances of the microbial community of the rhizosphere and the tobacco plants remarkably decreased after the *R. solanacearum* infection. Moreover, the gene abundances related to the nitrogen metabolism of the rhizosphere varied significantly, implying a correlation between *R. solanacearum* infection and the total nitrogen (TN) and ammonium nitrogen (NH_4_^+^-N) contents in the soil. All these results indicate that the *R. solanacearum* infection disturbed the microbiome structure and nitrogen metabolism in both the soil and the plants. This report will also discuss the complex network of interactions between the soil environment, the microbial community of the rhizosphere, and the endophytes after an infection of *R. solanacearum*. This understanding will help develop a novel strategy for preventing and controlling secondary disasters caused by plant pathogens through the management of nitrogen fertilizing techniques and the balance of microbial communities.

## Materials and Methods

### Experimental Design

The center of the selected experimental site was located in Huangdao District, Qingdao City, Shandong Province, China (119°7260′ E, 36°0196′ N). In 2020, the average precipitation in Qingdao City was 983.4 mm; the average temperature was 13.5°C; and the average sunshine hours were 2,407.2 h. According to whether *Ralstonia solanacerum* infected the plants in the experimental field, the soil plots that infected to the inside of the plants were set as the high disease group (STs), and the plots with no disease symptoms were set as the healthy group (CKs), respectively. The sites of CKs and STs were located in the same large field, and the field size was nearly 10,000 m^2^.

A total of four sections of plant roots, stems, leaves and inter-root soil were set up in the two groups, and nine samples were collected from each group, and three were randomly mixed and coded into one group. That is, each group had a total of three biological replicates. To ensure the accuracy of the experiment, all field management measures such as fertilizer application were kept the same for tobacco seedlings grown in both groups.

### Sample Collection

Samples were collected at nearly 90 days after tobacco transplanting in 2021. Two previously divided locations were selected to collect plant and soil samples. The plants were pulled from the soil, the root soil was shaken, collected in sample bags, immediately placed in a foam box filled with ice packs and dry ice and transported frozen to the laboratory, where the soil samples were immediately processed, passed through a 1 mm sieve, and separated into sterilized tubes and refrigerated at −80°C. The three parts of the plant, root, stem and leaves, were cut with sterilized scissors and collected in sample bags, placed in a foam box and transported to the laboratory for immediate processing of the plant samples, and the three parts of the root, stem and leaves were chopped with a sterile knife and placed in sterilized tubes and refrigerated at −80°C.

### Soil Nitrogen Content Analysis

The residues, such as plant roots, were filtered through a 1 mm sieve, mixed and divided equally into two parts, one part was shade dried and collected for reserve, and the other part was collected and frozen at −4°C for reserve. Soil total nitrogen (TN) was determined by Kjeldahl Apparatus (Kjeltec™ 8000) digestion and distillation of soil samples; soil ammonium nitrogen (NH_4_^+^-N) was determined by KCL leaching and indophenol blue colourimetric method; soil nitrate nitrogen (NO_3_^−^-N) was determined by phenol disulphonic acid colourimetric method. Soil organic carbon (SOC) was determined using the external heating method with potassium dichromate. The pH of the soil samples was measured using a pH meter. Determination of soil available potassium (AK) content using a flame photometer, ammonium acetate leaching method.

### Plant Nutrient Content Analysis

The plants were divided into three parts: root, stem and leaf to determine their nutrient contents. The plant samples were dried and crushed into powder form, and the amount of total phosphorus (TP) and total potassium (TK) in the three parts of the root, stem and leaves were measured using Optical emission spectrometer (OPTMA8000DV), and the content of trace elements iron (Fe) and magnesium (Mg) in the three parts of the plant were also determined.

### Soil Microbial Diversity Analysis

The main procedures included soil DNA extraction, PCR amplification, Illumina sequencing (MiSeq platform), sequence preprocessing and bioinformatics analysis. Soil DNA was extracted from soil microbial communities using the E.Z.N.A.^®^ SOIL DNA Kit (Omega Bio-Tek, Norcross, GA, United States). DNA extracts were measured on a 1% agarose gel and DNA concentration and purity were determined using a Nanodrop 2000 UV-Vis spectrophotometer (Thermo Scientific, Wilmington, United States). The bacterial 16S rRNA V3-V4 highly variable region was amplified using primers 338F (5′-ACT​CCT​ACG​GGA​GCA​GCA​GC-3′) and 806R (5′-GGACTACHVGGGTWTCTAAT-3′). The fungal ITS highly variable region was amplified using primers ITS1F (5′-CTT​GGT​CAT​TTA​GAG​GAA​GTA​A-3′) and ITS2R (5′-GCT​GCG​TTC​TTC​ATC​GAT​GC-3′). The bacterial PCR formal test was performed using TransGen AP221-02: TransStart Fastpfu DNA polymerase, 20 μl reaction system: 5 Fastpfu Buffer4 μL, 2.5 mm dNTPs2 μl, forward primer (5 μM) 0.8 μl, reverse primer (5 μM) 0.8 μl, Fastpfu ITS1F-ITSZR primer pair amplification PCR formal test using TaKaRaTaq DNA polymerase, 20 μl reaction system: 10x buffer 2 μl, 2.5 mM nucleotides 2 μl, forward primer (5 μM) 0.8 μl, reverse primer (5 μM) 0.8 μl, Fastpfu The procedure for bacterial 16S and fungal ITS amplification using a PCR instrument (ABI GeneAmp@9700) was as follows: pre-denaturation at 95°C for 3 min, followed by denaturation at 95°C for 30 s, annealing at 55°C for 30 s, extension at 72°C for 45 s, extension at 72°C for 10 min, and end at 10°C for a total of 30 cycles.

Purified amplicons were pooled in equimolar and paired-end sequenced (2 × 300) on an Illumina MiSeq platform (Illumina, San Diego, CA, United States). Barcode-tagged amplicons from different samples were mixed in equimolar concentration, and sent to the Majorbio Bio-Pharm Technology Co., Ltd. (Shanghai, China) for Miseq library construction and sequencing. The original fastq files were quality-filtered by Trimmomatic and merged by FLASH with the following criteria: 1) the reads were truncated at any site receiving an average quality score<20 over a 50 bp sliding window. 2) Sequences whose overlap being longer than 10 bp were merged according to their overlap with mismatch no more than 2 bp. 3) Sequences of each sample were separated according to barcodes (exactly matching) and Primers (allowing 2 nucleotide mismatching), and reads containing ambiguous bases were removed. Operational taxonomic units (OTUs) were clustered with 97% similarity cutoff using UPARSE (Edgar, 2013) with a novel “greedy” algorithm that performs chimera filtering and OTU clustering simultaneously. The taxonomy of each 16S rRNA gene sequence was analyzed by RDP Classifier algorithm against the Silva (SSU123) 16S rRNA database (Quast et al., 2012) using confidence threshold of 0.7. The raw reads were deposited in the NCBI Sequence Read Archive with the accession number of PRJNA788316 and are freely available at the NCBI (https://www.ncbi.nlm.nih.gov/bioproject/PRJNA788316/).

### Plant DNA Extraction and Amplification

After surface-sterilized, 0.5 g of tobacco tissue samples (in triplicate) were weighed, which were used for bacterial DNA extraction and analysis. The 16S ribosomal RNA gene variable region of plant endophytic bacteria was amplified using universal primers 799F (5′-AACMGGATTAGATACCCKG-3′) and 1193R (5′-ACG​TCA​TCC​CCA​CCT​TCC-3′), which could minimize contamination from chloroplast or mitochondrial 16 S rRNA genes of tobacco (Tian and Zhang, 2017). Low cycle count amplification was used; ensuring a uniform cycle count for each sample amplification. Samples were first randomly selected for pre-experimentation to ensure that the vast majority of samples at the lowest number of cycles would amplify the product at the right concentration, in order to adequately prepare all samples for the formal experiment. After the completion of the pre-test, the formal PCR test was performed using TransGen AP221-02: TransStart Fastpfu DNA Polymerase, 20 μl reaction system: 4 μl of 5 × FastPfu Buffer, 2 μl of 2.5 mM dNTPs, 0.8 μl of Forward Primer (5 μM) The PCR instrument (ABI GeneAmp^®^ 9700) was programmed as follows: 95°C for 3 min, number of cycles × (95°C for 30 s; 55°C for 30 s) C for 30 s; 55°C for 30 s; 72°C for 45 s), 72°C for 10 min and termination at 10°C. Two rounds of amplification were carried out, one with 27 cycles and the second with 13 cycles.

### Plant Endophytic Bacterial Community Analysis

The main procedures included Illumina sequencing (MiSeq platform), sequence preprocessing and bioinformatics analysis. The detailed procedures were described in the previously published paper (Du et al., 2015). Sequence processing and quality filtering, denoising, trimming, and merging of raw paired-end FASTQ files were performed using an improved dual-indexing approach and FLASH software to obtain clean tags. The chloroplast and mitochondrial DNA was eliminated from further analyses. The obtained clean tags were assigned into operational taxonomic units (OTUs) using USEARCH (v7.0.1090) at 97% identity clustering. The observed species index, Chao index, ACE index, Shannon index and Simpson index were determined using R software v3.1.1, which reflected the alpha diversity. The Shannon diversity and Chao1 richness were determined and principal coordinate analysis (PCoA) was performed using QIIME (v1.80). Heatmap and ternary plots were analyzed using R v3.1.1. All data were statistically analyzed using one-way analysis of variance. Tukey’s honestly significant difference test was used to separate means at *p* = 0.05. The above operations were performed at Majorbio Bio-pharm Technology Co., Ltd. (Shanghai, China). The raw reads were deposited in the NCBI Sequence Read Archive with the accession number of PRJNA788326. The processed sequences were clustered into operational taxonomic units (OTUs) with a 97% similarity using UPARSE (version 7.1). The species richness and diversity index were obtained using MOTHUR. The phylogenetic affiliation of each gene sequence was analysed using the RDP Classifier against the Silva (SSU115) with a confidence threshold of 70%. Hierarchical cluster analysis was performed using R software package. A Venn diagram was drawn to describe the similarities and differences among samples. The phenotypic traits of bacterial community were predicted with BugBase (http://bugbase.cs.umn.edu).

### Verification of the Nitrogen Metabolic Genes Using Absolute Quantitative PCR

The key functional genes of nitrogen cycling in soil were quantitatively analyzed. Including *nifH*, amoA, *arch-amoA*, *nirS*, *nirK*, *nosZ*, *narG*, *nxrA* and other genes. 5 min TM TA/Blunt-Zero CLoning Kit was used to construct standard plasmids containing target genes. The reaction system is 1 μl 5*TA/Blunt-Zero CLoning mix and 4 μl PCR product fragment. Primers used for gene amplification are shown in Supplementary Table S1.

The absolute quantitative PCR was carried out with Applied Biosystems 7500 Real-Time PCR System (Applied Biosystems, CA, United States) by using the SYBR Green I fluorescent dye detection in 20-μl volumes containing 10 μl of SYBR Premix Ex Taq (TaKaRa Biotech. Co, Japan), 2 μl of template, and 0.4 μl of both forward and reverse primers (10 mM each). The qPCR was performed by initially denaturing for 30 s at 95 C with subsequent cycling for 40 times with a 5 s denaturizing step at 95°C. The protocol was followed by a 34 s elongation/extension step at 60°C and with a melt curve analysis for 15 s at 95°C followed by 1 min at 60°C and finally for 15 s at 95°C. Melting curves were obtained based on a standard protocol and used to identify the characteristic peak of PCR product. Four independent technical replicates were used for each sample. And *t*-test analysis of the qRT-PCR results has been listed in Supplementary Figure S1.

### Statistical Analysis

Prism software was used to perform *t*-tests on soil nitrogen indicators. Soil microbial data and plant endogenous microbial data were analyzed using Uparse (version 7.0.1090), OTU clustering analysis of sequencing results and comparison of bacterial and archaeal ribosomal data in the Sliva database. Venn diagram analysis was performed using statistical tools in R. Species significance tests were performed using R Stats in Python and SciPy to obtain the genus level of significant differences between groups. Principal component analysis (PCA) and redundancy analysis (RDA) were performed using the R package. The Qiime software platform was used to calculate the abundance and beta diversity of each taxonomic microorganism. Evolutionary trees were constructed according to the approximately-maximum-likelihood phylogenetic trees using FastTree (version 2.1.3, http://www.microbesonline.org/fasttree/), and evolutionary trees were plotted using the evolutionary trees were plotted using the R language. PICRUSt (version 1.1.0, http://picrust.github.io/picrust/) was used for KEGG, COG and Pfam function prediction of 16S sequences. Species differences and functional differences were analyzed using the R package doing wilcoxon rank sum tests.

## Results

### Differences in Soil Physicochemical Properties Between the Healthy Group and the Diseased Group

The physicochemical properties of the soil after *R. solanacearum* infection presented significant differences (Table 1). The total nitrogen (TN) of the diseased group (ST) was remarkably higher than that of the healthy group (CK) (*p* = 0.0003, ***). The highest measured TN results for the two groups were 604.35 mg kg^−1^ and 485.53 mg kg^−1^, respectively. In contrast, the ammonium nitrogen (NH_4_^+^-N) content of the CK group was higher than that of the ST group. However, nitrate nitrogen (NO_3_^−^-N) showed a similar content in both groups. In addition to the nitrogen content, the pH values, the available potassium (AK), and the soil organic matter (SOM) all demonstrated significant differences between the two groups, in which the pH values of the CK group were lower than those of the ST group (*p* = 0.0041, **), and the SOM content presented the inverse relationship (*p* = 0.0019, **). Meanwhile, as an important nutrient index for stress resistance, the highest AK content in the CK group was 571.87 mg kg^−1^ whereas the ST group’s AK content ranged between320.81 and 431.19 mg kg^−1^ (*p* = 0.0001, ***).

**TABLE 1:** Soil physicochemical indicators in the soil.

Sample	TN (mg/kg)	pH	NO_3_^−^-N (mg/kg)	NH_4_^+^-N (mg/kg)	SOM (g/kg)	SOC (g/kg)	AK (mg/kg)
CK1	330.06 ± 51.60	5.93 ± 0.05	15.75 ± 1.24	4.11 ± 0.30	7.59 ± 0.22	4.40 ± 0.13	482.25 ± 4.43
CK2	485.53 ± 28.96	5.92 ± 0.09	16.48 ± 0.64	4.97 ± 0.41	7.51 ± 0.34	4.36 ± 0.20	565.51 ± 28.14
CK3	390.13 ± 0.37	5.87 ± 0.04	14.59 ± 1.47	2.86 ± 0.69	8.11 ± 0.68	4.70 ± 0.40	571.87 ± 67.49
ST1	599.49 ± 95.19	6.12 ± 0.09	14.56 ± 0.58	1.80 ± 0.02	7.07 ± 0.34	4.10 ± 0.20	431.19 ± 26.77
ST2	491.01 ± 142.09	6.13 ± 0.02	14.17 ± 0.10	2.31 ± 0.19	6.32 ± 0.13	3.67 ± 0.07	443.97 ± 9.22
ST3	604.35 ± 35.48	5.92 ± 0.10	16.76 ± 1.31	1.40 ± 0.01	7.29 ± 0.26	4.23 ± 0.15	320.81 ± 20.98

Note: the two groups of soil samples were CK (healthy soil) and ST (diseased soil), respectively; TN, total nitrogen in soil; pH, pH in soil; NO_3_^−^-N, soil nitrate nitrogen; NH_4_^+^-N, soil ammonium nitrogen; SOM, soil organic matter; SOC, soil organic carbon; AK, soil available potassium.

### Differences of Microbial Community Structure in the Soil Between the Healthy Group and the Diseased Group

**FIGURE 1:**
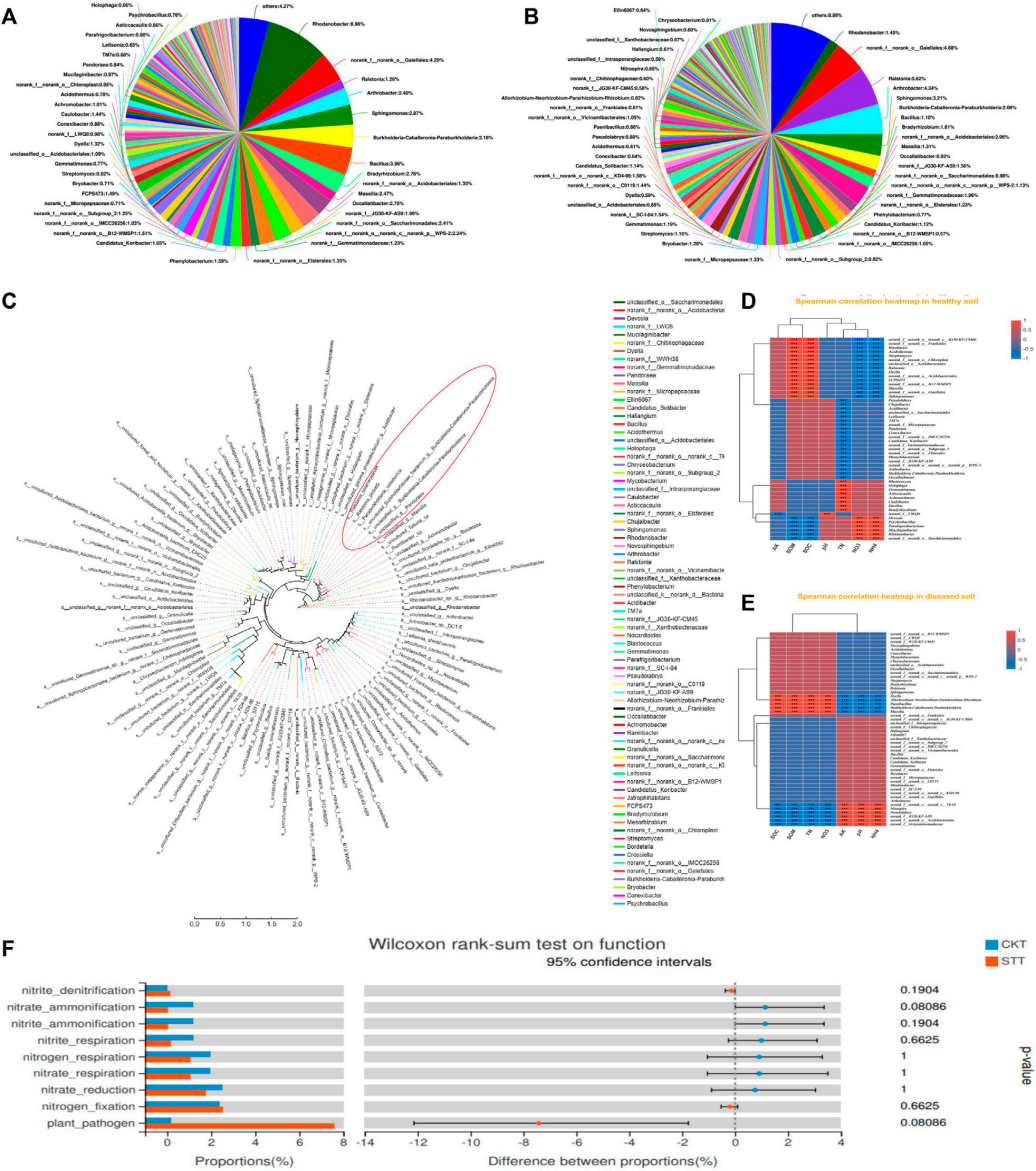
Microbiology community of soil samples analysis: **(A)** the composition of soil bacterial community in healthy group, **(B)** the composition of soil bacterial community in diseased group; **(C)** the phylogenetic tree of soil bacterial diversity; **(D)** the correlation analysis between the microbial genera and soil physicochemical properties (TN, pH, NO_3_, NH_4_, SOM) in the CK group; **(E)** the correlation analysis between the microbial genera and soil physicochemical properties (TN, pH, NO_3_, NH_4_, SOM) in the ST group; **(F)** the functional intergroup test of soil samples.

The microbial communities presented significant differences between the CK and ST groups. In the CK group, bacterial phyla such as *Rhodanobacter* (8.96%), *galellates* (4.20%), *Bacillus* (3.96 %), *Burkholderia-caballeronia-paraburkholderia* (3.18%), *Sphingomonas* (2.87%), *and Arthrobacter* (2.40%) dominated the microbiome in soil, in which the *Ralstonia* phylum was also present and only accounted for 1.26% (Figure 1A). However, the dominant bacterial species in the ST group varied greatly, including *Ralstonia* (5.62%), *galellates* (4.68%), *Arthrobacter* (4.34%), *Sphingomonas* (3.21%), and *Burkholderia-caballeronia-paraburkholderia* (2.08%). The proportion of the *Rhodanobacter* phylum decreased to1.45%but the proportion of the *Ralstonia* phylum significantly increased, which was the most abundant taxon in the ST group and was directly related to the disease onset of the tobacco plant (Figure 1B). Thus, the infection of *R. solanacearum* might have resulted in a decrease in the abundance of some dominant phyla in the CK group. In general, the relationship between some species and soil physicochemical properties was not clear (Supplementary Figure S2). Therefore, CKs and STs were respectively associated with environmental factors to find the key species. Moreover, the dominant species correlated with the soil physicochemical properties in both the CK and ST groups (Figures 1D,E). In the CK group, the abundance of *Ralstonia* was negatively correlated with NH_4_^+^-N and NO_3_^−^-N and positively correlated with SOM and SOC. However, *Rhodanobacter* presented the inverse correlations with the physicochemical properties of the soil to that of *Ralstonia*. In contrast to the healthy soil, the interactions between the diseased soil bacteria and the physicochemical properties changed considerably at the genus level. *Ralstonia* showed an insignificant positive correlation with NO_3_^−^N contents and its negative correlation with NH_4_^+^-N decreased. Similarly, the positive correlation between *Rhodanobacter* and NH_4_^+^-N also decreased after *R. solanacearum* infection, and its correlation with soil NO_3_^−^N was insignificantly negative. the correlations between *Sphingomonas* and the physicochemical properties of the soil were the same as those between *Ralstonia* and the physicochemical properties of the soil in both the CK and ST groups (Supplementary Figure S1). All these results indicate that the infection of *R. solanacearum* not only changes the structure of the microbial community of the soil but also disturbs the correlations between the microbial community of the rhizosphere and the physicochemical conditions of the soil. Phylogenetic analysis was performed to explore the species closely related to *R. solanacearum* in genetic relationships. The results showed that *Burkholderia* was the closest species related to *R. solanacearum*, and the other dominant phyla, *Rhodanobacter* and *Arthrobacter,* had distant genetic relationships with *R. solanacearum* (Figure 1C). Close relatives might assist in *R. solanacearum* infection, while distant relatives might inhibit disease onset. Furthermore, the distantly related bacterial genera showed diverse interactions with the physicochemical properties of the soil, such as changes in the nitrogen content, which is consistent with previous reports that soil nitrogen cycling has an important effect on both plant growth and rhizosphere microbial survival (Hu et al., 2020).Furthermore, the differentiation of the two groups on soil nitrogen metabolic functions and plant pathogenicity was analyzed (Figure 1F). The functions of nitrite denitrification, nitrogen fixation, and plant pathogens were stronger in the ST group than in the CK group. However, most of the nitrogen cycling processes driven by rhizosphere microorganisms in the healthy soil, including the ammonification of nitrate and nitrite, the respiration of nitrogen, nitrate, and nitrite, and nitrate reduction, were all stronger than those in the diseased soil. The results indicate that the infection disturbed the microbial community of the soil and caused an imbalance in the nitrogen metabolism in soil.

### Differences of Nutrient Contents in the Plants Between the Healthy Group and the Diseased Group

The nutrient contents in the tobacco plant’s roots, stem, and leaves were measured (Table 2). Overall, the content of total potassium (TK) in the stems and leaves were higher than that in roots in both the CK and ST group. Moreover, the contents of TK in the roots of the ST group were slightly higher than in the roots of the CK group (*p* = 0.0029, **). Unlike TK, there were significant differences in the content of total phosphorus (TP) between the CK and ST groups. The TP contents in the roots, stems, and leaves of the ST group were all lower than those in the CK group. Similarly, the contents of total nitrogen (TN) in the ST group were also significantly lower than those in the CK group. Furthermore, the contents of iron (Fe) and magnesium (Mg) showed differentiation between the two groups. The contents of Fe in the roots and leaves of the CK group were lower than those in the ST group, with an inverse trend for the stems. Similar to TP, the contents of Mg within the whole plant from the ST group were lower than those in the CK group. All these results imply that *R. solanacearum* infection can result in significant differences in the nutrient contents of plants.

**TABLE 2:** Nutrient contents in the plant.

Sample	TK (mg/kg)	TP (mg/kg)	Fe (mg/kg)	Mg (mg/kg)	TN (g/kg)
CKG	9.20 ± 0.31	0.76 ± 0.02	1.57 ± 0.09	2.16 ± 0.12	65.80 ± 16.20
CKJ	17.77 ± 0.31	0.98 ± 0.08	0.40 ± 0.01	3.89 ± 0.17	158.82 ± 4.61
CKY	16.70 ± 0.40	1.22 ± 0.11	0.68 ± 0.01	9.69 ± 0.17	340.23 ± 5.52
STG	10.94 ± 0.35	0.48 ± 0.05	2.07 ± 0.13	1.45 ± 0.04	41.96 ± 0.29
STJ	16.39 ± 0.24	0.53 ± 0.03	0.16 ± 0.01	1.41 ± 0.02	37.22 ± 2.31
STY	25.31 ± 0.37	0.51 ± 0.01	1.92 ± 0.02	6.28 ± 0.07	141.60 ± 5.11

Note: CKG represents the root sample of the healthy tobacco plant; STG represents the root sample of the diseased tobacco plant; CKJ represents the stem sample of the healthy tobacco plant; STJ represents the stem sample of the diseased tobacco plant; CKY represents the leaf sample of the healthy tobacco plant; STY represents the leaf sample of the diseased tobacco plant.TN represents the total nitrogen nutrient content of tobacco plants; TP represents total phosphorus nutrient content of tobacco plants; TK represents total k nutrient content of tobacco plants; Fe represents the iron content of tobacco plants; Mg represents the magnesium content of tobacco plants.

### *R. solanacearum* Infection Raised the Changes in Community Structure of Plant Endophytes

**FIGURE 2:**
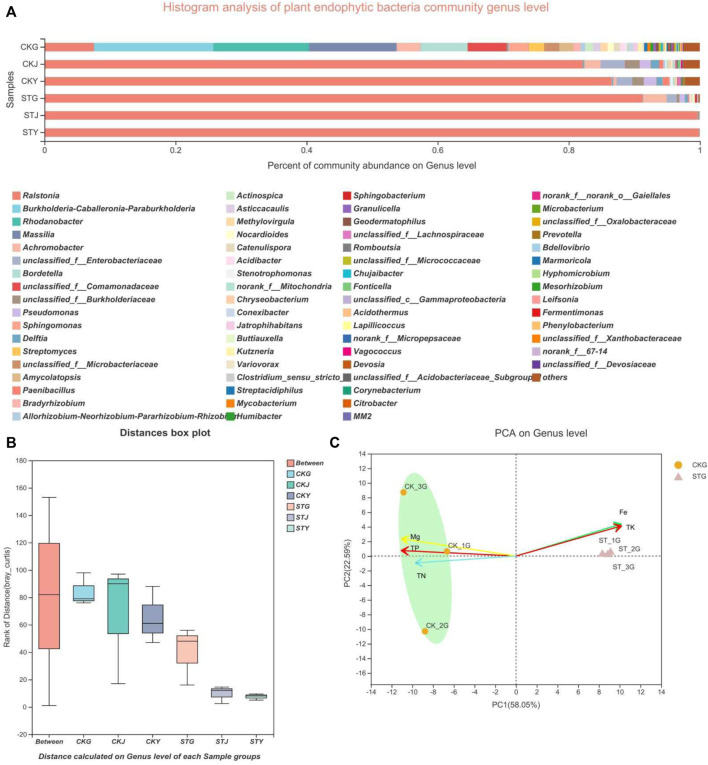
Microbiology community of plant samples analysis: **(A)** plant endophytic bacteria community structure, **(B)** plant endophytic bacteria sample grouping analysis, **(C)** plant endophytic bacteria community principal component analysis.

The rhizosphere environment of soil-borne diseases can greatly impact the status of plants, including the diversity and community structure of their endophytes. As a pathogen capable of infecting more than 200 crops, *R. solanacearum* can also influence the community composition of plant endophytes. To verify whether endophytic bacteria are directly affected by soil diseases, not just by the plant’s nutrient content, the diversity of the endophytic bacteria in healthy and diseased tobacco plants was analyzed. By comparing the roots, the stem and the leaves, it was found that the bacterial community structures in the healthy plants were more complicated and diversified than those in the diseased plants. The phyla *Rhodanobacter*, *Massilia*, and unclassified-f-*Comamonadaceae* showed different proportions between the two groups. Meanwhile, the abundance of the endophytic phyla in the roots, stems, and leaves of healthy tobacco plants was higher than that of diseased plants. Although the *Ralstonia* genus dominated the endophytic community in both the healthy and diseased plants, the diversity of bacteria in the roots, stems, and leaves in the healthy group was higher than that in the diseased group, and the number of dominant bacterial genera was different (Figures 2A,B). *R. solanacearum* is a soil-borne disease so the diversity within the roots will be looked at in closer detail. The results showed that *Rhodanobacter* and *Massilia* existed in the roots of healthy plants but were not found in the roots of diseased plants. And that there were abundant microbes associated with nitrogen metabolism present in the healthy roots, such as *Paraburkholderia*, *Bordetella*, *Achromobacter*, unclassified-f-*Comamonadaceae*, and *Sphinngomonas* (Figure 2A). The roots with more diverse endophytes showed a similarity in the endophytic phyla, however, the PCA revealed that the microbial species included in the same phylum were significantly different from each other (Figure 2C). In brief, the *R. solanacearum* infection also significantly affected the endophytic flora of tobacco plants.

**FIGURE 3:**
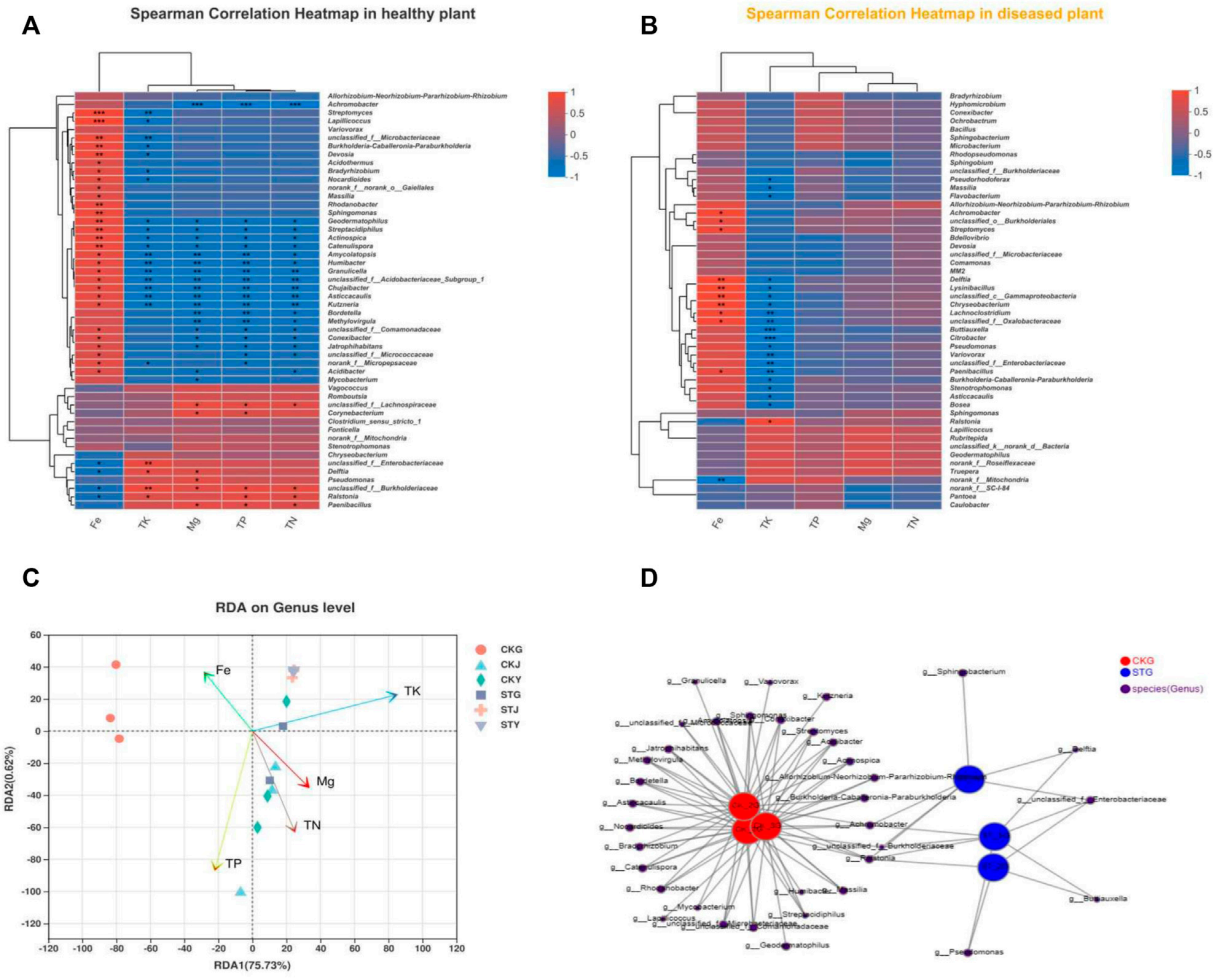
Analysis on the relationship between endophytic bacteria and plant nutrient content: Heatmap of correlation analysis between endophytes and plant nutrients of healthy **(A)** and diseased **(B)** tobacco plants, redundancy analysis of nutrient content and correlation of tobacco plants **(C)**, network analysis of bacterial diversity in roots of healthy group (CKs) and disease group after RS infection (STs) **(D)**.

Plant nutrient contents are directly related to crop quality and yield. However, it is unclear how plant nutrients correlate with the plant endophytic community after *R. solanacearum* infection in tobacco. Here, the relationship between endophytic bacterial genera and plant nutrient content was analyzed. The results showed that the contents of TP, TN, and Mg showed more significant correlations with the endophytes in healthy plants, with almost no correlation in diseased plants (Figures 3A,B). Although the content of Fe also presented a similar correlation with the endophytic community after *R. solanacearum* infection, the corresponding significance decreased compared to that of the healthy group. This result indicated that some association exists between the variation in the endophytic community and plant nutrients. *Ralstonia* was positively correlated with the plant’s TP, TK, and TN, in contrast to the negative correlation with the Fe content in healthy tobacco plants. Nevertheless, most endophytic bacteria in the healthy tobacco plants were negatively correlated with TP, TK, and TN but positively correlated with the content of Fe in the plants, which was the inverse to that of *Ralstonia* (Figure 3A). Unlike in healthy plants, the correlations between TN, TP, Fe, and *Ralstonia* were reduced in the diseased plants. Interestingly, the TK content of the plants still showed a significant positive correlation with *Ralstonia* after its infection (Figure 3B). All these results indicate that the *R. solanacearum* infection had no influence on the TK content, while the TN and TP were significantly affected. Soil-borne disease infection starts in the plant’s rhizosphere; therefore, more attention has been given to the microbial community within the roots. RDA demonstrated that there was a significant difference in the microbial community composition of the healthy and diseased tobacco roots. The correlation between healthy root’s endophytes and tobacco Fe content was stronger, while the correlation between diseased root endophytes and tobacco TN/Mg nutrients was stronger (Figure 3C). Even more notable is that the endophytic community in the roots had a higher richness than that in the stems and leaves regardless of the healthy or diseased samples. Based on the network analysis of the bacterial diversity within the roots, *Burkholderia*, *Achromobacter,* and *Ralstonia* existed in both the healthy and diseased plants (Figure 3D). However, the healthy roots showed a higher degree of differentiation in species diversity than the diseased roots, in which *Delftia* and *Buttiauxella* were present. The results indicate that the variation in the endophytic community might interact with the absorption and utilization of the plant’s nutrients, both of which were influenced by the *R. solanacearum* infection.

**FIGURE 4:**
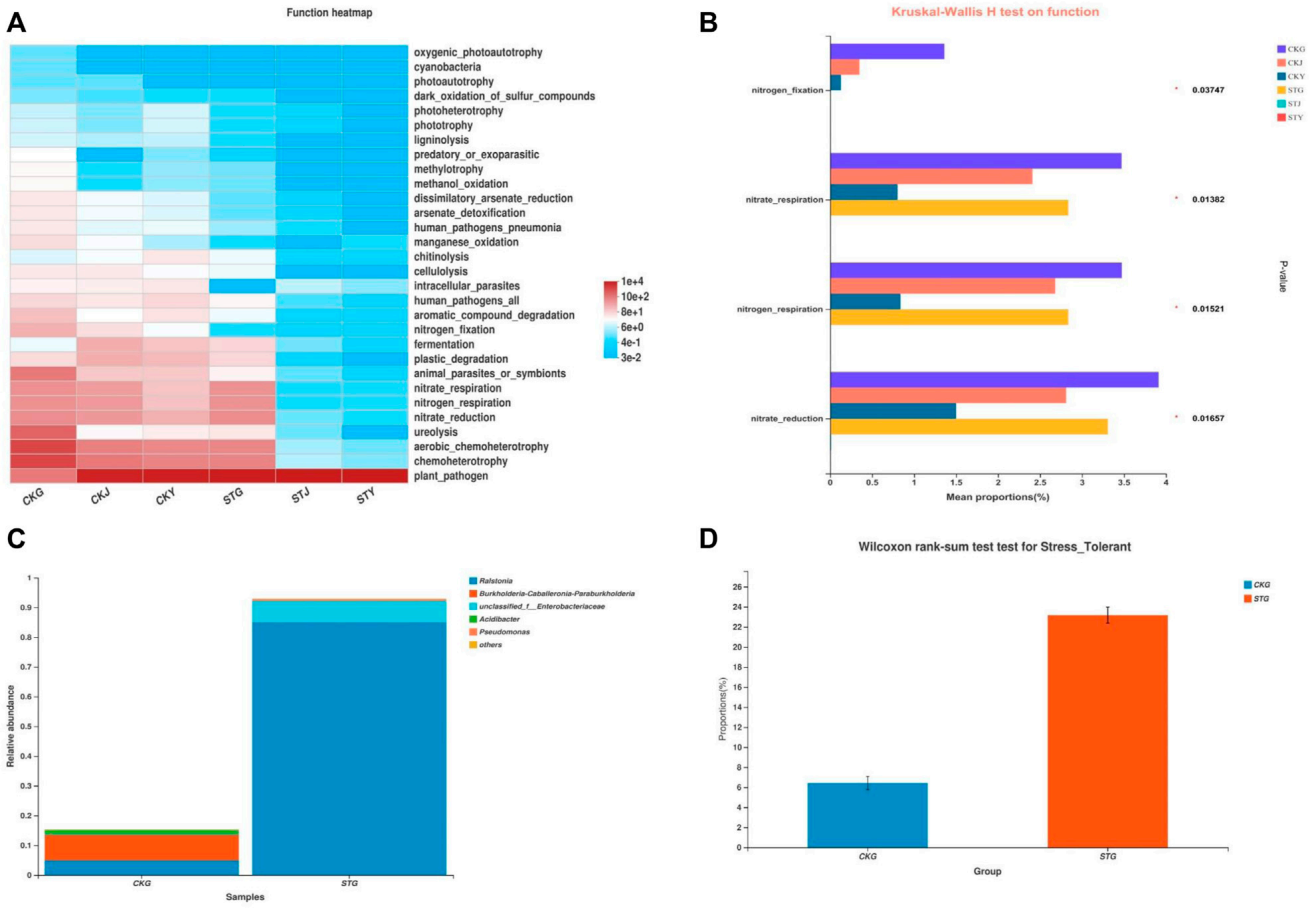
Functional and phenotypic differences induced by RS infection: Prediction of nitrogen metabolism-related functions of endophytic bacteria diversity in tobacco plants **(A)** and differentiation test of function prediction **(B)**, analysis of the phenotypic contribution of different species in different groups to stress tolerance **(C)** and differentiation test **(D)**.

The heatmap analysis of the predicted gene functional groups included in the endophytic microbes indicated that the intensity of the nitrogen metabolism in the roots, stems, and leaves of the diseased plants, including nitrate reduction, nitrate respiration, nitrogen respiration, nitrogen fixation, and ureolysis, decreased significantly compared to that in the healthy plants, especially in the stems and leaves (Figure 4A). The decreased chemoheterotrophic function might imply a reduction in the metabolism and utilization of nutrients after *R. solanacearum* infection. In addition, the nitrogen metabolism intensity of the healthy roots was significantly stronger than that of the stems and leaves, which was also reflected in the diseased plants (Figure 4B). These were generated from the differentiation of the endophytic communities in the diseased and healthy roots. Meanwhile, *Ralstonia* accounted for a greater proportion of the endophytic community in the diseased root than in the healthy root, indicating that a greater tolerance could prevent the pathogen from being eliminated by plant autoimmunity and thus allow for the onset of wilt disease in the tobacco crop (Figures 4C,D).

### The Nitrogen Metabolism-Related Genes Varied in Diseased Soil Compared to Those in Healthy Soil

The nitrogen cycle within the soil is driven by soil microbes and thus the diversity and abundance of these microbes are important for analyzing soil sample characteristics. To analyze the change in microbial biomass caused by *R. solanacearum* infection, the microbial biomass carbon (MBC), the microbial biomass nitrogen (MBN), and the response ratio between the MBC and the MBN (MBC: MBN) of the soil were analyzed (Supplementary Table S2), which reflects information on the overall microbial community structure (Shen et al., 2016; Hua et al., 2021). The abundance of the *R. solanacearum* pathogen in the diseased soil led to significantly lower MBC and MBN compared to those in the healthy soil. In addition, the original organic matter in the soil, the soil microbes, and other organisms release a variety of organic matter into the soil (Culliney, 2013). While changing the structure of the microbial community of the soil, the abundance of *R. solanacearum* might also compete for nutrients with other microbes. Furthermore, the MBC: MBN ratio in the healthy soil was 7.31 and that in the diseased soil was 5.47, which indicates that the microbial community structure of the soil changed after the pathogen infection. In general, the MBC: MBN ratio of bacteria, actinomycetes, and fungi were approximately 5:1, 6:1, and 10:1, respectively (Xiao et al., 2015). Therefore, the higher MBC: MBN in the healthy soil demonstrated that bacteria were not the only dominant microbe; fungi and actinomycetes also accounted for significant proportions, where these different species interacted with each other and maintained a balance in the soil microecology. The dominant bacterium, *R. solanacearum* pathogen, resulted in the reduction of the MBC: MBN ratio and disrupted the harmony between the soil bacteria, fungi, and actinomycetes, facilitating the process of infection.

**FIGURE 5:**
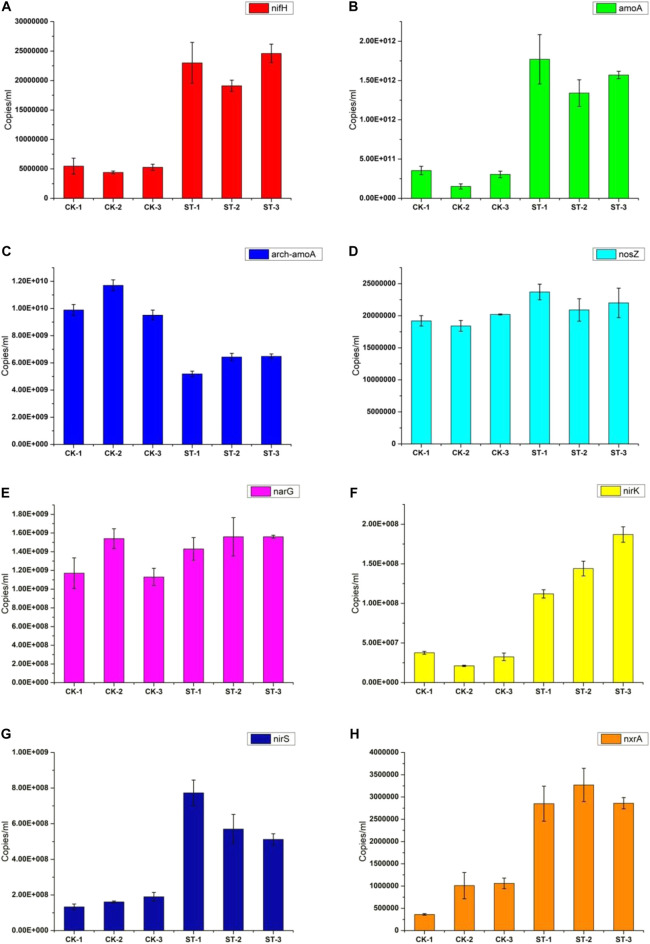
Quantitative PCR results of key genes in soil nitrogen cycle. **(A–H)** are for the genes of *nifH*, *amoA*, *arch-amoA*, *nosZ*, *narG*, *nirK*, *nirS*, and *nxrA*, respectively.

N cycling in soil ecosystems consists of several metabolic processes, mainly N fixation, nitrification, and denitrification. In these processes, many microbial nitrogen metabolism functional genes are involved (Levy-Booth et al., 2014). To clearly explain the differences in the soil nitrogen metabolism caused by the *R. solanacearum* infection, the absolute abundances of the nitrogen metabolic genes (*nifH*, *amoA*, *arch-amoA*, *nirK*, *nirS*, *nxrA*, *narG,* and *nosZ*) in the healthy and diseased soils were determined using absolute quantitative PCR. As shown in Figure 5, the abundance of *nifH* in the diseased soil, as a representative of a nitrogen fixation gene, was much higher than that in the healthy soil, indicating that the abundance of nitrogen-fixing microbes in the root soil increased significantly during the *R. solanacearum* infection process. At the same time, the *amoA* gene involved in soil nitrification also presented a higher abundance in the diseased soil, which can convert NH_4_^+^ to NH_2_OH. In addition, the *arch-amoA* gene had a lower abundance in the diseased soil, indicating that the *R. solanacearum* infection resulted in a reduction in archaeal abundances, followed by a decrease in the nitrification process in which archaea participate. Moreover, the abundances of *nirK* and *nirS* genes, which are included in the denitrification process were higher in the diseased soil. The *nxrA* gene, which is responsible for the conversion of NO_2_^−^ to NO_3_^−^, also had a higher abundance in the diseased soil. However, the *narG* and *nosZ* genes, which are involved in the nitrification and denitrification processes, respectively, showed no significant differences. Meanwhile, the copy numbers of the *nirK* and *nirS* genes were much higher than that of the *nxrA* gene in all the samples, implying that the intensity of the denitrification process was stronger than that of the nitrification process. In summary, most of the nitrogen metabolic genes in the rhizosphere varied significantly, resulting in a stronger denitrification process after the *R. solanacearum* infection. The differences in the nitrogen metabolic genes in the healthy and the diseased soils were correlated with the differential microbial community and the physicochemical properties of the soil, all of which were influenced by the *R. solanacearum* infection.

## Discussion

In this work, the impact of the R. solanacearum infection on the physicochemical properties of the soil, the microbial community structure of the rhizosphere, and the endophytic microbiome composition were studied. Moreover, the responses to the *R. solanacearum* infection of key functional genes involved in the nitrogen metabolism in soil were explored. The results showed that the microbial communities in both the soil and plants of the diseased tobacco niche were significantly different from those in the healthy tobacco, and the two phyla *Delftia* and *Buttiauxella* coexisted with the *Ralstonia* pathogen in both the healthy and diseased samples. It was also verified that the genes related to nitrogen metabolism, including *nifH*, *amoA*, *arch-amoA*, *nirK*, and *nxrA*, presented distinct abundances in the diseased and healthy soils. With the combination of these gene expressions, the diseased soil presented lower contents of NH_4_^+^-N compared to the healthy soil, which was correlated with the change of the microbial community of the rhizosphere after the *R. solanacearum* infection, and stronger denitrification processes were achieved in the diseased soil. Additionally, the correlations between the physicochemical properties of the soil and the microbial community also varied greatly. Together, these results indicate that the *R. solanacearum* infection significantly disturbed the microbiome structure within the whole tobacco niche (the rhizosphere soil the tobacco roots, the stems, and the leaves) as well as the nitrogen metabolism in soil.

The infection of *R. solanacearum* requires extensive colonization in the planting soil first, which is naturally influenced by the physicochemical properties of the soil. Therefore, indicators including pH and the contents of carbon, nitrogen, and phosphorus have been reported to be directly related to the *R. solanacearum* pathogen (Li et al., 2017; Wei et al., 2018; Li P et al., 2021). Wei et al. found that the water-soluble nitrogen concentration increased during *R. solanacearum* infection in tomato plants (Wei et al., 2018). Nitrate availability has been reported to affect *R. solanacearum* pathogen virulence by modulating virulence gene expression during root attachment and colonization in the initial infection stage. Moreover, this pathogen can utilize the nitrogen metabolism for respiring and generating energy within the low-oxygen and/or NO-rich microenvironments of the plant’s xylem vessels (Dalsing and Allen, 2014). The content and metabolism of nitrogen exhibited stronger correlations with the *R. solanacearum* infection compared to the other soil physicochemical properties. In this work, the higher total nitrogen (TN) content and lower ammonium nitrogen (NH_4_^+^-N) content in the diseased group indicated that the *R. solanacearum* infection remarkably disturbed nitrogen metabolism within the rhizosphere. In addition, the nutrient contents (TK, TP, TN, Fe, and Mg) included in the roots, the stems, and the leaves also changed after the *R. solanacearum* infection and were directly related to the nutrient contents within the rhizosphere. Notably, the trace metal Mg, as an important component of chlorophyll, significantly decreased after the *R. solanacearum* infection, which might also be a reason for leaf yellowing and wilting. Meanwhile, the Fe content which is an important component of ferredoxin which participates in the electron transport of photosynthesis as well as in nitrate reduction and nitrogen fixation changed after the pathogen infection implying that Fe content and nitrogen metabolism might function synergistically. As reported, iron homeostasis is important for host-pathogen interactions by signaling iron-regulated host immune responses when plant pathogens infect (Verbon et al., 2017). Furthermore, Gu et al. demonstrated that competition for iron via secreted siderophore molecules is a good predictor of microbe–pathogen interactions and plant protection, providing a promising strategy to use siderophore-mediated interactions as a tool for microbiome engineering and pathogen control (Gu et al., 2020).

In addition to the changes in the physicochemical properties of the soil, the association between the plant pathogens and the changes in the structure of the microbial community of the rhizosphere have also been observed. The microbial diversity decreased after the *R. solanacearum* infection, with changes in the composition of the microbial community of the rhizosphere. *Ralstonia* showed the inverse correlation with NO_3_^−^-N in the healthy and diseased groups. As the most dominant phylum in the healthy samples, the proportion of *Rhodanobacter* remarkably decreased after the infection, one species of which, *Rhodanobacter denitrificans,* is capable of the denitrification process (Prakash et al., 2012). Together, these results indicate that the potential association between *R. solanacearum* infection and nitrogen metabolism. Additionally, some taxa, such as *Sphingomonas* and *Sphingomonas melonis*, which can increase disease resistance against bacterial pathogens (Matsumoto et al., 2021), presented the same correlations with the physicochemical properties of the soil as *Ralstonia* in both the healthy and diseased groups, implying that there is a synergistic relationship between the two taxa during pathogen infection. The phylum *Delftia* was discovered in the diseased samples and has been reported to play a helping role in the infection process of the pathogen (Hu et al., 2020; Li M et al., 2021). Moreover, another phylum, *Buttiauxella*, also showed a similar role as *Delftia* in this study. After the pathogen infected plant tissues, the diversity of the endophytic microbial community and the abundance of species decreased. Notably, *Actinobacteroia* was abundant in the roots of healthy tobacco plants, which have resistance genes and are capable of producing antibiotics. *Actinobacteroia* might also participate in the inhibition of *R. solanacearum* infection within healthy tobacco plants (Yuliar et al., 2015; Fatahi-Bafghi, 2019). It is worth mentioning that the *R. solanacearum* pathogen existed in both healthy and diseased samples with different abundances and proportions, indicating that it is difficult for the single *R. solanacearum* pathogen to result in disease onset. Taken together, it can be deduced that the pathogen infection process interacts with the physicochemical properties of the soil, the plant nutrients, the composition of the host-associated rhizosphere and endophytic microbiome communities and that all these factors interact with each other.

The nutrient cycles in plant cultivation, especially the nitrogen cycle, are mostly driven by microbes (Liu et al., 2010; Nelson et al., 2015). Plants can absorb nitrate and nitrite from the soil, which are the key compounds within the soil nitrogen cycling. It has been verified that the availability of nitrate is important for the expression of virulence genes in *R. solanacearum* during its colonization process in tomatoes (Dalsing et al., 2015). In this study, the genes associated with nitrogen metabolism within the soil varied significantly after the *R. solanacearum* infection. These results demonstrate that the nitrogen-fixing capacity increased in the diseased soil, while the nitrification process from NH_4_^+^ to NH_2_OH charged by *amoA* in bacteria was significantly stronger than that charged by *arch-amoA* in archaea. However, the high concentration of denitrifying bacteria presented in the diseased soil prevented further nitrification of NH_2_OH, leading to nitrogen N losses by forming NO and NO_2_ which are released into the atmosphere as greenhouse gasses and thus contribute to environmental pollution. The high intensity of the *nxrA* gene in the diseased soil may be due to the resistance effects of soil bacteria to environmental stress (Xiao et al., 2017; Pinto et al., 2020). The copies of the *nxrA* gene in both the diseased and healthy soil were much lower than those of the remaining nitrogen metabolic genes, probably because nitrite is easily oxidized and does not require too many microbes to participate in the reaction process. Nevertheless, this is not nearly enough to compensate for the loss of nitrogen in the diseased soil. Conversely, the lower abundances of *nirK* and *nirS* genes maintained the normal nitrification process in the healthy soil. Therefore, the intensity of nitrogen cycling in the diseased soil was lower than that in the healthy soil, and the changed nitrogen metabolic genes resulted in a stronger denitrification process after the *R. solanacearum* infection.

**FIGURE 6:**
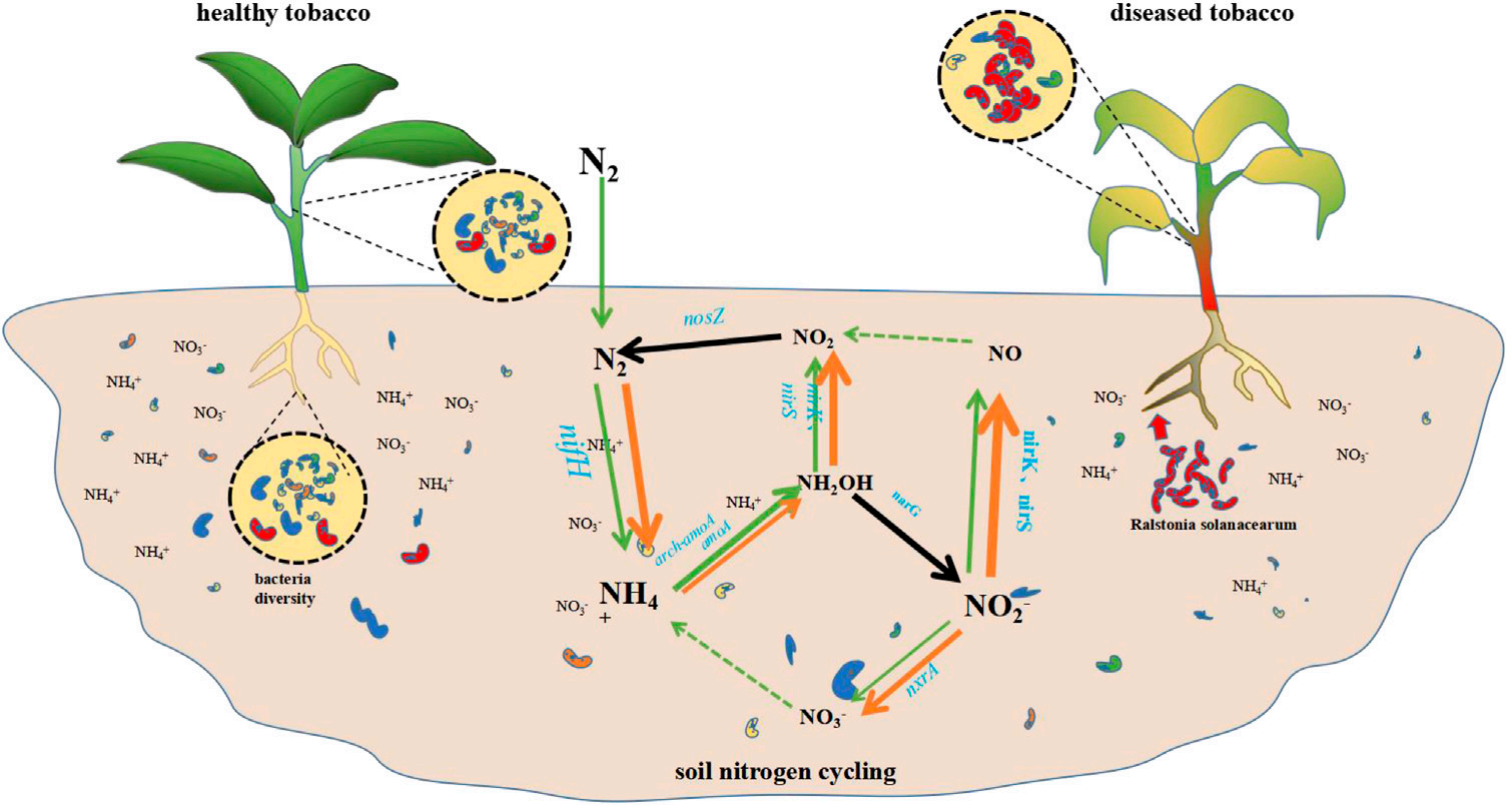
*R. solanacearum* infection disturbed microbiome structure throughout the whole niche and soil nitrogen metabolism. The short rods in red represent *R. solanacearum* pathogen, the short rods in other colors represent the other microbes in soil or in plant. The arrows involved in the nitrogen cycling represent the nitrogen metabolic reactions in soil (orange, diseased soil; green, healthy soil), and the thickness of the lines represent the absolute abundances of the genes. The black arrows represent no differences between the diseased and healthy soil.

A summary diagram of the effects on the physicochemical properties of the soil, the plant’s nutrients, and the composition of the host-associated the microbial community of rhizosphere and endophytic caused by the *R. solanacearum* infection in the tobacco plants can be seen in Figure 6. As shown, the infection resulted in a decrease in the diversity of the microbiome structure throughout the whole niche, including the rhizosphere, the plant roots, the stems, and the leaves. Meanwhile, the contents of NH_4_^+^ decreased as a result of the variation in the nitrogen metabolism, which was caused by the synergic action of the nitrogen metabolism genes with different absolute abundances within the soil nitrogen cycling.

To effectively prevent secondary disasters in crop cultivations, several suggestions have been proposed as follows. First, as this work concludes, a large amount of nitrogen in the planting soil might result in a decrease in the microbial biomass of the soil and changes in the microbial community structure of the rhizosphere. The application of nitrogen fertilizers can have negative effects on the diversity of bacteria and protozoa in soil (Ge et al., 2021). As a result of this work, the *R. solanacearum* infection leaded to a higher content of total nitrogen, which might be one reason for the reduction in the microbial diversity. In addition, our previous study verified that a reduction in nitrogen fertilizer was beneficial to tobacco growth. Together, these results raise the potential of reducing fertilizing, especially nitrogen, as a potential strategy for inhibiting pathogen infection. Second, the balance of the microbiome in soil is significantly important during crop growth. As shown by the above results, the *R. solanacearum* infection disturbed the diversity of the microbial structure of the soil, which could possibly be recovered by adding microbial inoculants or biological organic fertilizer. Third, it has been described that *R. solanacearum* infection can led to remarkable decreases in the MBC: MBN ratio, indicating a disruption in the balance between microbes, bacteria, fungi, and actinomycetes. Therefore, the intentional addition of some corresponding fungi and actinomycetes could harmonize the microbial biomass. Meanwhile, some fungi and actinomycetes can release antibiotics to resist the infection of pathogens (Jakubiec-Krzesniak et al., 2018; Michela et al., 2018). All these suggestions can provide constructive farmland management strategies for the prevention of soil-borne secondary diseases in agricultural production.

## Conclusion

Bacterial wilt infection raised significant effects on microorganism of rhizosphere, soil environment, plant and the correlation among these factors. Besides, the copies of microbial nitrogen metabolism genes within the rhizosphere significantly varied after the *R. solanacearum* infection, resulted in a stronger denitrification process. Together, bacterial wilt infection destroyed the microbial community structure and soil nitrogen metabolism of the whole tobacco niche. These results suggest that the application and management of nitrogen fertilizers and a balance of microbial community can be promising strategies for preventing and controlling soil-borne secondary disasters caused by plant pathogens.

## Data Availability

The datasets presented in this study can be found in online repositories. The names of the repository/repositories and accession number(s) can be found below: https://www.ncbi.nlm.nih.gov/, PRJNA788326;https://www.ncbi.nlm.nih.gov/, PRJNA788326.
