# Supplementary figures and images for: Ralstonia solanacearum Infection Disturbed the Microbiome Structure Throughout the Whole Tobacco Crop Niche as Well as the Nitrogen Metabolism in Soil
Source: Front Bioeng Biotechnol. 2022 Jun 21;10:903555. doi: 10.3389/fbioe.2022.903555 (PMC9253565; doi:10.3389/fbioe.2022.903555)

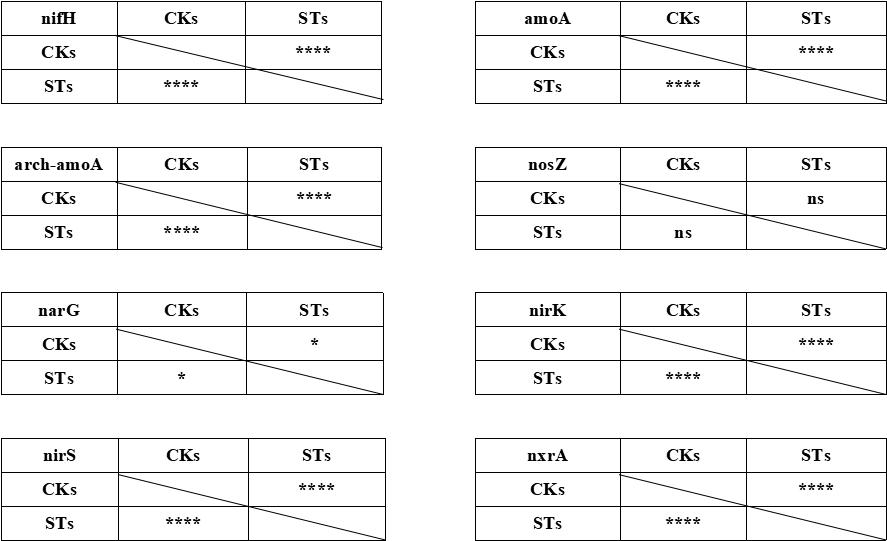

Supplement: Supplementary file 1 [file Image2.JPEG]
