# Supplementary material for: Ralstonia solanacearum Infection Disturbed the Microbiome Structure Throughout the Whole Tobacco Crop Niche as Well as the Nitrogen Metabolism in Soil
Source: Front Bioeng Biotechnol. 2022 Jun 21;10:903555. doi: 10.3389/fbioe.2022.903555 (PMC9253565; doi:10.3389/fbioe.2022.903555)
Supplement: Supplementary file 2 [file Table1.doc]

**Table S1.** Primers for N-cycling genes and the qPCR cycling conditions for each primer set.

| Target gene | Primers | | Annealing temperatures °C | Reference |
| --- | --- | --- | --- | --- |
| *nif*H | PolR | ATSGCCATCATYT C RCCGGA | 55 | (Poly et al., 2001) |
|  | PolF | TGCGAYCCSAA RGCBGACTC |  |  |
| archaeal *amo*A | Arch-amoAF | STAATGGTCTGGCTTAGACG | 55 | (Francis et al., 2005) |
|  | Arch-amoAR | GCGGCCATCCATCTGTATGT |  |  |
| bacterial *amo*A | AmoA-1F | GGGGTTTCTACTGGTGGT | 61 | (Rotthauwe et al., 1997) |
|  | AmoA-2R | CCCCTCKGSAAAGCCTTCTTC |  |  |
| *nxr*A | F1370 F1 | CAGACCGACGTGTGCGAAAG | 60 | (Poly et al., 2008) |
|  | F2843 R1 | TCCACAAGGAACGGAAGGTC |  |  |
| *nar*G | *narG* 1960 F | TAYGTSGGCCARGARAA | 55 | (Philippot et al., 2002) |
|  | *narG* 2650 R | TTYTCRTACCABGTBGC |  |  |
| *nir*S | Cd3aF | GTSAACGTSAAGGARACSGG | 58 | (Throback et al., 2004) |
|  | R3cd | GASTTCGGRTGSGTCTTGA |  |  |
| *nir*K | F1aCu | ATCATGGTSCTGCCGCG | 58 | (Hallin and Lindgren, 1999) |
|  | R3Cu | GCCTCGATCAGRTTGTGGTT |  |  |
|  | Ns-norB-710R | ATGCGYGGSAWRTAGAAGWAMAMSA |  |  |
| *nos*Z | nosZF | CGYTGTTCMTCGACAGCCAG | 56 | (Di et al., 2014) |
|  | nosZ1662R | CGSACCTTSTTGCCSTYGCG |  |  |

Di, H.J., Cameron, K.C., Podolyan, A., and Robinson, A. (2014). Effect of soil moisture status and a nitrification inhibitor, dicyandiamide, on ammonia oxidizer and denitrifier growth and nitrous oxide emissions in a grassland soil. *Soil Biol Biochem*. doi: 10.1016/j.soilbio.2014.02.011.

Francis, C.A., Roberts, K.J., Beman, J.M., Santoro, A.E., and Oakley, B.B. (2005). Ubiquity and diversity of ammonia-oxidizing archaea in water columns and sediments of the ocean. *Proc Natl Acad Sci U S A* 102(41)**,** 14683-14688. doi: 10.1073/pnas.0506625102.

Hallin, S., and Lindgren, P.E. (1999). PCR detection of genes encoding nitrite reductase in denitrifying bacteria. *Appl Environ Microbiol* 65(4)**,** 1652-1657. doi: 10.1128/AEM.65.4.1652-1657.1999.

Philippot, L., Piutti, S., Martin-Laurent, F., Hallet, S., and Germon, J.C. (2002). Molecular analysis of the nitrate-reducing community from unplanted and maize-planted soils. *Appl Environ Microbiol* 68(12)**,** 6121-6128. doi: 10.1128/AEM.68.12.6121-6128.2002.

Poly, F., Monrozier, L.J., and Bally, R. (2001). Improvement in the RFLP procedure for studying the diversity of nifH genes in communities of nitrogen fixers in soil. *Res Microbiol* 152(1)**,** 95-103. doi: 10.1016/s0923-2508(00)01172-4.

Poly, F., Wertz, S., Brothier, E., and Degrange, V. (2008). First exploration of Nitrobacter diversity in soils by a PCR cloning-sequencing approach targeting functional gene nxrA. *FEMS Microbiol Ecol* 63(1)**,** 132-140. doi: 10.1111/j.1574-6941.2007.00404.x.

Rotthauwe, J.H., Witzel, K.P., and Liesack, W. (1997). The ammonia monooxygenase structural gene amoA as a functional marker: molecular fine-scale analysis of natural ammonia-oxidizing populations. *Appl Environ Microbiol* 63(12)**,** 4704-4712. doi: 10.1128/aem.63.12.4704-4712.1997.

Throback, I.N., Enwall, K., Jarvis, A., and Hallin, S. (2004). Reassessing PCR primers targeting nirS, nirK and nosZ genes for community surveys of denitrifying bacteria with DGGE. *FEMS Microbiol Ecol* 49(3)**,** 401-417. doi: 10.1016/j.femsec.2004.04.011.
