# Supplementary material for: Ralstonia solanacearum Infection Disturbed the Microbiome Structure Throughout the Whole Tobacco Crop Niche as Well as the Nitrogen Metabolism in Soil
Source: Front Bioeng Biotechnol. 2022 Jun 21;10:903555. doi: 10.3389/fbioe.2022.903555 (PMC9253565; doi:10.3389/fbioe.2022.903555)
Supplement: Supplementary file 4 [file Table2.doc]

**Table S2. Detection of microbial biomass carbon (MBC), microbial biomass nitrogen (MBN), and the response ratio between MBC and MBN (MBC/MBN) of the soil.**

|  | CK | ST |
| --- | --- | --- |
| MBC | 182.48±19.75 | 75.27±15.30 |
| MBN | 27.84±9.27 | 13.03±0.52 |
| MBC/MBN | 7.31±3.53 | 5.47±0.21 |
